# Impact of Waist Circumference and Body Mass Index on Risk of Cardiometabolic Disorder and Cardiovascular Disease in Chinese Adults: A National Diabetes and Metabolic Disorders Survey

**DOI:** 10.1371/journal.pone.0057319

**Published:** 2013-03-08

**Authors:** Xuhong Hou, Juming Lu, Jianping Weng, Linong Ji, Zhongyan Shan, Jie Liu, Haoming Tian, Qiuhe Ji, Dalong Zhu, Jiapu Ge, Lixiang Lin, Li Chen, Xiaohui Guo, Zhigang Zhao, Qiang Li, Zhiguang Zhou, Guangliang Shan, Zhaojun Yang, Wenying Yang, Weiping Jia, for the China National Diabetes and Metabolic Disorders Study Group

**Affiliations:** 1 Department of Endocrinology and Metabolism, Shanghai Jiao Tong University Affiliated Sixth People’s Hospital, Shanghai Diabetes Institute, Shanghai Clinical Center for Diabetes, Shanghai, China; 2 Department of Endocrinology and Metabolism, Chinese People’s Liberation Army General Hospital, Beijing, China; 3 Department of Endocrinology and Metabolism, Sun Yat-sen University Third Hospital, Guangzhou, China; 4 Department of Endocrinology and Metabolism, Peking University People’s Hospital, Beijing, China; 5 Department of Endocrinology and Metabolism, First Affiliated Hospital, Chinese Medical University, Shenyang, Liaoning, China; 6 Department of Endocrinology and Metabolism, Shanxi Province People’s Hospital, Taiyuan, Shanxi, China; 7 Department of Endocrinology and Metabolism, West China Hospital, Sichuan University, Chengdu, Sichuan, China; 8 Department of Endocrinology and Metabolism, Xijing Hospital, Fourth Military Medical University, Xi’an, Shaanxi, China; 9 Department of Endocrinology and Metabolism, the Affiliated Drum Tower Hospital of Nanjing University Medical School, Nanjing, Jiangsu, China; 10 Department of Endocrinology and Metabolism, Xinjiang Uygur Autonomous Region’s Hospital, Urmqi, Xinjiang, China; 11 Department of Endocrinology and Metabolism, Fujian Provincial Hospital, Fuzhou, Fujiang, China; 12 Department of Endocrinology and Metabolism, Qilu Hospital of Shandong University, Jinan, Shandong, China; 13 Peking University First Hospital, Beijing, China; 14 Department of Endocrinology and Metabolism, Henan Province People’s Hospital, Zhengzhou, Henan, China; 15 Department of Endocrinology and Metabolism, Second Affiliated Hospital of Harbin Medical University, Harbin, Heilongjiang, China; 16 Department of Endocrinology and Metabolism, Xiangya Second Hospital, Changsha, Hunan, China; 17 Department of Epidemiology and Statistics, Institute of Basic Medical Sciences, Chinese Academy of Medical Sciences, School of Basic Medicine, Peking Union Medical College, Beijing, China; 18 Department of Endocrinology and Metabolism, China–Japan Friendship Hospital, Beijing, China; NIDDK/NIH, United States of America

## Abstract

**Background:**

We updated the prevalence of obesity and evaluated the clinical utility of separate and combined waist circumference (WC) or body mass index (BMI) category increments in identifying cardiometabolic disorder (CMD) and cardiovascular disease (CVD) risk in Chinese adults.

**Methods and Findings:**

46,024 participants aged ≥20 years, a nationally representative sample surveyed in 2007–2008, were included in this analysis. Taking the cutoffs recommended by the Chinese Joint Committee for Developing Chinese Guidelines (JCDCG) and the Working Group on Obesity in China (WGOC) into account, the participants were divided into four WC and four BMI groups in 0.5-SD increments around the mean, and 16 cross-tabulated combination groups of WC and BMI. 27.1%, 31.4%, and 12.2% of Chinese adults are centrally obese, overweight, or obese according to JCDCG and WGOC criteria. After adjustment for confounders, after a 1-SD increment, WC is associated with a 1.7-fold or 2.2-fold greater risk of having DM or DM plus dyslipidemia than BMI, while BMI was associated with a 2.3-fold or 1.7-fold higher hypertension or hypertension plus dyslipidemia risk than WC. The combination of WC and BMI categories had stronger association with CMD risk, i.e., the adjusted ORs (95% CI) of having DM, hypertension, and dyslipidemia for the combined and separate highest WC and BMI categories were 2.19 (1.96–2.44) vs 1.88 (1.67–2.12) and 1.12 (0.99–1.26); 5.70 (5.24–6.19) vs 1.51 (1.39–1.65) and 1.69 (1.57–1.82); and 3.73 (3.42–4.07) vs 2.16 (1.98–2.35) and 1.33 (1.25–1.40), respectively. The combination of WC and BMI categories was more likely to identify individuals with lower WC and lower BMI at CVD risk, even after the effects of CMD were controlled (all *P*<0.05).

**Conclusion:**

Central obesity, overweight, and obesity are epidemic in Chinese adults. The combination of WC and BMI measures is superior to the separate indices in identifying CMD and CVD risk.

## Introduction

Cardiovascular disease (CVD) is the leading cause of morbidity and mortality in the world [1] and is the most prevalent disease affecting the health of the Chinese population [2]. It is anticipated that cardiometabolic disorder (CMD) and CVD together will become an even more serious public health burden as the prevalence of obesity, the most important cardiometabolic risk factor [3]–[6], continues to rise rapidly in China, as it has over the past two decades [7], [8].

Although they are not perfect measures of body fat, body mass index (BMI) and waist circumference (WC) are the most practical indices for identifying obesity in clinical practice and epidemiologic research. It is known that specific BMI values can reflect different body compositions across genders and races; eg, in some Asian populations a given BMI value indicates a higher percentage of body fat than in European populations [9]–[11]. Because WC reflects a measure of central fat distribution, while BMI reflects a combination of both fat mass and lean mass, some researchers have argued that WC is a better indicator of an adverse metabolic profile than BMI [12], [13]. Additionally, several studies have reported that WC increases much faster than BMI over the same time period [14], [15], so the question of whether BMI or WC is more predictive of CMD and CVD risk in Chinese adults becomes significant.

As part of the China National Diabetes and Metabolic Disorders Study, the goal of this analysis was to assess the prevalence of obesity in Chinese adults and to evaluate the clinical utility of separate and combined WC and BMI categories and category increments in identifying the risk of CMD and CVD in this population. To our knowledge, there has not been any systematic assessment based upon a large representative sample reported for Chinese adults.

## Methods

### Study Design and Population

This current paper is one of a series of reports on different themes from the China National Diabetes and Metabolic Disorders Study, which was a representative cross-sectional survey of Chinese adults from June 2007 to May 2008. This study was designed to provide up-to-date prevalence figures for diabetes mellitus (DM) and related metabolic disorders in Chinese adults. Three papers from the study have been published and have been referred to in the present manuscript [2], [16], [17]. The first paper provided data on the prevalence and associated risk factors of diabetes and prediabetes, while the second paper mainly reported the morbidity and associated risk factors of non-fatal cardiovascular diseases. Note that this paper also presented the prevalence of classic cardiovascular risk factors, including overweight or obesity as identified by WHO criteria (Overweight: 25 kg/m^2^≤ BMI <30 kg/m^2^; obesity: BMI ≥30 kg/m^2^). The third paper provided levels of serum lipids and lipoproteins and estimated the prevalence, awareness, treatment, and control of hypercholesterolemias.

Our current report focused on evaluating the clinical utility of separate and combined waist circumference (WC) and body mass index (BMI) categories within the same increment in assessing presence of cardiometabolic and cardiovascular diseases in Chinese adults, while at the same time, we also reported the prevalence of overweight, obesity and central obesity identified by China diagnostic criteria.

The multi-stage sampling process used in the survey, including sampling size, sampling scheme, and several other features, has been described in detail previously [16]. In the first stage, 11 provinces (Guangzhou, Liaoning, Shanxi, Sichuan, Shaanxi, Jiangsu, Fujian, Shandong, Henan, Heilongjiang, Hunan), 1 autonomous region (Xinjiang), and 2 municipalities (Beijing and Shanghai) were involved in this survey, based on geographic region, population size, and economic status. In the the next two stages, 52 city districts, 24 county seats, and 56 rural townships were selected according to economic status. In the fourth stage, 2 street-level districts or rural villages (about 500–1,000 households) were randomly selected from each urban city district (or county seat) and rural township, respectively. In the fifth stage, target populations were stratified by gender and age-group, and individuals were randomly selected from each stratum. Individuals who had lived in their current residence for 5 years or longer were eligible to participate. At the conclusion of the sampling process, a total of 54,240 residents ≥20 years old were selected from the general population and invited to participate in this survey. Overall, 47,325 individuals (18,976 men and 28,349 women) participated; 46,024 of these (18,326 men and 27,698 nonpregnant women) with complete data on fasting plasma glucose (FPG), 2-hour postprandial plasma glucose (2hPPG), WC, and BMI were included in this study.

This study was approved by the institutional review boards of 17 participating institutions, which are the institutional review board of Shanghai Jiao Tong University Affiliated Sixth People’s Hospital, the institutional review board of Chinese People’s Liberation Army General Hospital, the institutional review board of Sun Yat-sen University Third Hospital, the institutional review board of Peking University People’s Hospital, the institutional review board of First Affiliated Hospital of Chinese Medical University, the institutional review board of Shanxi Province People’s Hospital, the institutional review board of West China Hospital of Sichuan University, the institutional review board of Xijing Hospital of Fourth Military Medical University, the institutional review board of the Affiliated Drum Tower Hospital of Nanjing University Medical School, the institutional review board of Xinjiang Uygur Autonomous Region’s Hospital, the institutional review board of Fujian Provincial Hospital, the institutional review board of Qilu Hospital of Shandong University, the institutional review board of Peking University First Hospital, the institutional review board of Henan Province People’s Hospital, the institutional review board of Second Affiliated Hospital of Harbin Medical University, the institutional review board of Xiangya Second Hospital, and the institutional review board of China–Japan Friendship Hospital. Written informed consent was obtained from each participant before the survey. The 17 institutional review boards’ approvals covered every participant in the study.

### Blood Sample Collection and Laboratory Assessments

An oral glucose tolerance test was performed on all subjects. After subjects underwent at least 10 hours of overnight fasting, venous blood samples were drawn at 0, 30, and 120 minutes following ingestion of a 75-gram oral glucose load (for participants without a prior diagnosis of DM) or ingestion of a steamed bun containing approximately 80 g of complex carbohydrates (for participants with a self-reported history of DM).

Plasma glucose, serum cholesterol, and triglyceride levels were determined by enzymatic assay at the clinical biochemical laboratories of each province. All laboratory measurements met a standardization and certification program. Serum insulin levels were measured by a radioimmunoassay, and homeostatic model assessment of insulin resistance (HOMA-IR) was calculated using the formula HOMA-IR = fasting serum insulin (FINS, mIU/L)×FPG (mmol/L)/22.5 [18].

### Questionnaire

Trained staff administered a standardized questionnaire to each subject. Demographic data, lifestyle, smoking/drinking habits, family history, and medical history were collected during the survey.

For smoking and drinking habits, the subjects were divided into two categories (according to the information at the time of the survey): current smokers/drinkers and non-current smokers/drinkers. Current smokers were defined as those who had smoked ≥1 cigarette/day for at least 1 year. Current drinkers were defined as those who had consumed ≥30 g of alcohol/week on average for at least 1 year. Regular leisure-time physical activity was defined as participation in moderate or vigorous activity for 30 minutes or more per day at least 3 days a week. Educational level was also recorded and categorized into three groups: low (illiteracy, primary, and secondary education), medium (high school education) and high (college or university education). The questionnaire included family history of obesity, DM, hypertension, dyslipidemia, coronary heart disease (CHD), and stroke for first-degree relatives (biological mother, father, brothers, or sisters).

### Physical Examination

Blood pressure, body weight, and height were measured according to standard protocol [19]. BMI was calculated as weight over height squared (kg/m^2^). WC was measured at the horizontal plane between the inferior costal margin and the iliac crest on the mid-axillary line.

### Diagnostic Criteria

Central obesity was defined as WC ≥90 cm in men and ≥85 cm in women according to the Chinese Joint Committee for Developing Chinese Guidelines (JCDCG) Guidelines on Prevention and Treatment of Dyslipidemia in Adults (2007) [20]. Overweight and obesity were respectively identified as 24 kg/m^2^≤ BMI <28 kg/m^2^ and BMI ≥28 kg/m^2^ according to the Working Group on Obesity in China (WGOC) (2002) [21].

The MetS was defined according to the criteria established by the Chinese Joint Committee for Developing Chinese Guidelines on Prevention and Treatment of Dyslipidemia in Adults (JCDCG). The JCDCG-Mets was defined as three or more of the following abnormalities: 1. Central obesity (WC ≥90 cm for men and ≥85 cm for women); 2. Elevated TG (TG ≥1.7 mmol/L), or specific treatment for this lipid abnormality; 3. Reduced HDL-C (HDL-C <1.03 mmol/l), or specific treatment for this lipid abnormality; 4. Elevated BP (BP≥130/85 mmHg or current treatment for hypertension), or previously diagnosed hypertension; and 5. Elevated plasma glucose (FPG ≥6.1 mmol/L or 2 h PG ≥7.8 mmol/L) or previously diagnosed DM [20].

Cardiometabolic disorder (CMD) is defined as a clustering of disorders which includes DM, hypertension, and dyslipidemia (elevated TG, reduced HDL-C, and elevated LDL-C), but not obesity. DM was defined as FPG ≥7.0 mmol/L or 2 hPPG ≥11.1 mmol/L, or having been previously diagnosed with DM and receiving therapy [22].

Hypertension was diagnosed as systolic blood pressure (SBP) ≥140 mmHg or diastolic blood pressure (DBP) ≥90 mmHg, or having been diagnosed with hypertension and receiving antihypertensive therapy [23].

Dyslipidemia was defined as (1) elevated triglycerides (TG): TG ≥1.7 mmol/L, or drug treatment for this lipid abnormality; (2) reduced high-density lipoprotein cholesterol (HDL-C): HDL-C <1.03 mmol/L in men and <1.29 mmol/L in women; (3) elevated low-density lipoprotein cholesterol (LDL-C): LDL-C ≥3.37 mmol/L; or (4) use of cholesterol-lowering medication [24].

Non-fatal CVD (non-fatal CHD and non-fatal stroke) was determined according to a patient’s self-report. Incident of non-fatal CHD events were identified by a history of hospitalization for myocardial infarction or a surgical history of coronary balloon angioplasty, or coronary stent implantation or coronary artery bypass graft surgery. Non-fatal stroke events were identified by a history of language or physical dysfunction continuing for more than 24 h and ischemic or hemorrhagic stroke. These diagnostic criteria are wholly consistent with our previously published paper [2].

### Separate and Combined WC and BMI Groups

For men and women, the mean (SD) WC and BMI were 85.5 (10.5) cm and 79.4 (10.2) cm, and 24.5 (3.6) kg/m^2^ and 23.9 (3.7) kg/m^2^, respectively. Taking into account the gender-specific mean (SD) of each of the indices and the cut-offs recommended by JCDCG and WGOC, the participants were divided into four WC and four BMI groups: WC Group I (WC <85 cm in men and <80 cm in women), WC Group II (85–90 cm in men and 80–85 cm in women), WC Group III (90–95 cm in men and 85–90 cm in women), and WC Group IV (≥95 cm in men and ≥90 cm in women); BMI Group I (BMI <24 kg/m^2^), BMI Group II (24–26 kg/m^2^), BMI Group III (26–28 kg/m^2^), and BMI Group IV (≥28 kg/m^2^), respectively. These WC and BMI categories were cross-tabulated to form 16 combination WC and BMI (WC * BMI) groups.

These categories allowed us to evaluate the clinical utility of separate and combined WC and BMI cut-off points, at 0.5-SD increments (∼2 kg/m^2^ for BMI and 5 cm for WC) above the mean (BMI: 24 kg/m^2^; WC: 85 cm for men and 80 cm for women), in predicting risk for adverse CMD and CVD outcomes.

### Statistical Methods

Descriptive statistics were presented as mean with a 95% confidence interval (CI) or proportion (95% CI). Differences in mean were tested by *t*-test, and differences in proportion were tested by chi-square test. Linear trends for sex- and age-specific mean or proportion were respectively tested using ANOVA linear test (polynomial) or Chi-Square test for Linear-by Linear Association. Differences in proportion between men and women were tested using multivariable logistic regression analysis (adjusted for age and the interaction of region * gender).

Considering the study sampling scheme and differences between the sample surveyed and the total population, the prevalence data was corrected for several features of the survey [16] and was weighted to represent Chinese adults (≥20 years old) according to Chinese population data in 2006. The number of overweight and obese people was estimated according to standardized prevalence and the size of the Chinese population in 2006. Standard errors (SEs) were calculated according to the complexities of the survey design, as previously described [16].

Binary or multinominal multivariable logistic regression was conducted to assess the association of separate and joint WC and BMI categories with CMD and CVD using the Entry method; adjusted odds ratios (ORs) and the 95% CIs are given.

The dependent variables were CMD (DM, hypertension, elevated TG, reduced HDL-C, elevated LDL-C, or dyslipidemia) and CVD (CHD, stroke, and CVD) in binary logistic regression, and the dependent variables were the category variable of different CMD combinations with the group without any CMD as the referent in multinominal logistic regression.

**Figure 1:**
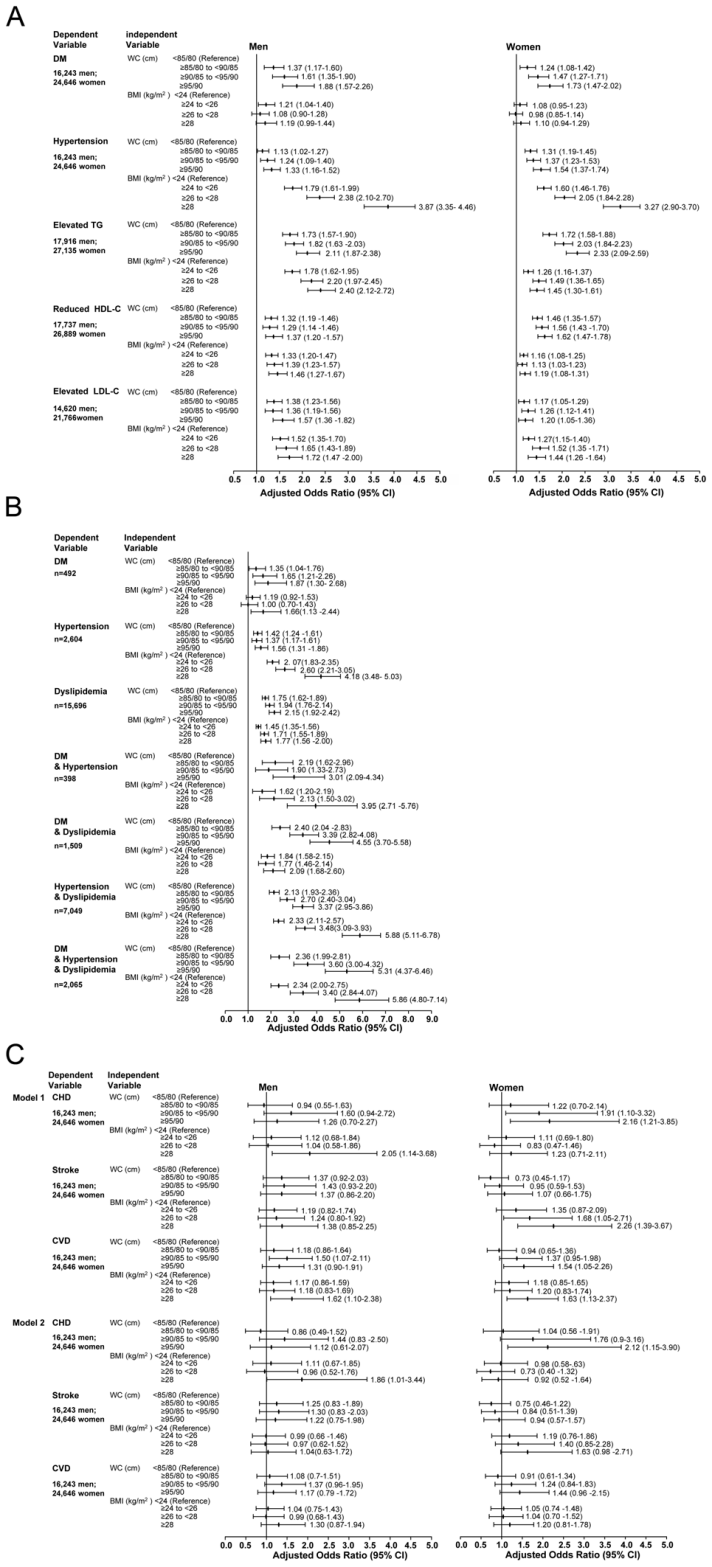
Associations of waist circumference/BMI categories with CMD and CVD. Binary or multinominal multivariable logistic regression was conducted to assess the association of separate WC and BMI categories with CMD (Figure 1A and Figure 1B) and CVD (Figure 1C) using the Entry method; adjusted odds ratios (ORs) and the 95% confidence intervals (CIs) are given. The dependent variables were CMD (DM, hypertension, elevated TG, reduced HDL-C, elevated LDL-C) and CVD (CHD, stroke, and CVD) in binary logistic regression, and the dependent variables were the category variable of different CMD combinations with the group without any CMD as the referent in multinominal logistic regression. The independent variables were mutually adjusted WC and BMI categories. Adjustment variables included the basic confounders (age, education levels, smoking status, drinking status, physical activity) and family history of diseases (identified as the dependent variables) in **Figure 1A** **,** **Figure 1B** **, and** **Figure 1C** **(**Model 1**)**. In **Figure 1C** (Model 2), CMD (DM, hypertension, and dyslipidemia) were also considered as confounders besides the adjustment variables above mentioned. Abbreviations: BMI, body mass index (calculated as weight in kilograms divided by the square of the height in meters); CHD, coronary heart disease; CI, confidence interval; CVD, cardiovascular disease; DM, diabetes mellitus; HDL-C, high-density lipoprotein cholesterol; LDL-C, high-density lipoprotein cholesterol; OR, odds ratio; TG, triglycerides; WC, waist circumference.

**Figure 2:**
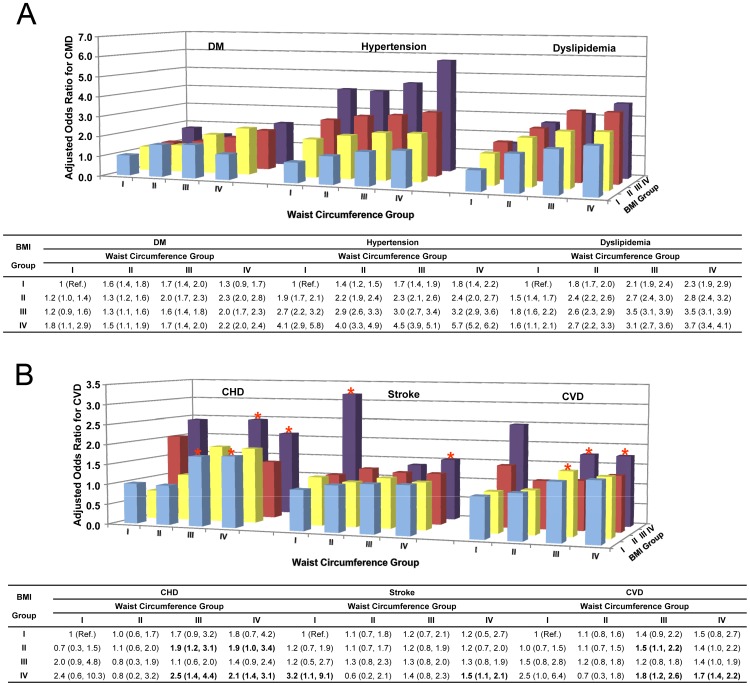
ORs for CMD and CVD among the combination groups of WC and BMI categories. Binary multivariable logistic regression was conducted to assess the association of combined WC and BMI categories with CMD **(** **Figure 2A** **)** and CVD **(** **Figure 2B** **)** using the Entry method and the adjusted odds ratios (ORs) and the 95% CIs were given. The dependent variables were CMD (DM, hypertension, and dyslipidemia) and CVD (CHD, stroke, and CVD).The independent variables were the combination group of WC and BMI categories. Adjustment variables included the basic confounders (gender, age, education levels, smoking status, drinking status, physical activity) and family history of diseases (the disease in accordance with the dependent variables) in **Figure 2A**. In **Figure 2B**, CMD (DM, hypertension, and dyslipidemia ) were also considered as confounders besides the adjustment variables in **Figure 2A**. *P<0.05 was asterisked only in **Figure 2B** **.** Abbreviations: BMI, body mass index (calculated as weight in kilograms divided by the square of the height in meters); CHD, coronary heart disease; CI, confidence interval; CVD, cardiovascular disease; DM, diabetes mellitus; OR, odds ratio; TG, triglycerides; WC, waist circumference.

The independent variables were mutually adjusted WC and BMI categories, shown in Figure 1, or joint WC and BMI categories, shown in Figure 2.

Adjustment variables included age, education level, smoking status, drinking status, physical activity, and family history of disease (identified as the dependent variables). Additionally, CMD (DM, hypertension, and dyslipidemia) and gender were also considered as confounding factors as appropriate.

The statistical analyses were performed using SUDAAN version 10 (RTI International, Research Triangle Park, NC, USA) and SPSS version 15.0 (SPSS Inc, Chicago, IL, USA). A value of *P*<0.05 (two-tailed) was designated as statistically significant. In addition, in view of the large sample size in our research, a higher level of significance *P*<0.01 (two-tailed) was adopted to explain the results in Tables 1 and 2.

**Table 1:** Characteristics of 46,024 Participants by Gender and Region Type.

Characteristic	Mean (95% CI)a,c				*P* value^d^			
	Men (n = 18,326)		Women (n = 27,698)		Urban vs Rural		Men vs Women	
	Urban (n = 11,355)	Rural (n = 6,971)	Urban (n = 18,001)	Rural (n = 9,697)	In Men	In Women	In Urban	In Rural
Age (years)	45.0 (44.9–45.1)	44.8 (44.7–44.9)	44.7 (44.6–44.8)	44.7 (44.6–44.8)	0.022	0.834	<0.001	0.355
Waist circumference (cm)	85.5 (85.2–85.8)	81.8 (81.4–82.2)	77.9 (77.7–78.2)	78.0 (77.7–78.3)	<0.001	0.753	<0.001	<0.001
BMI (kg/m^2^)	24.6 (24.5–24.7)	23.6 (23.4–23.7)	23.5 (23.4–23.6)	23.4 (23.2–23.5)	<0.001	0.126	<0.001	0.026
FPG (mmol/L)	5.39 (5.34–5.44)	5.22 (5.18–5.27)	5.30 (5.20–5.40)	5.16 (5.11–5.20)	<0.001	0.011	0.102	0.035
2 h PG (mmol/L)	7.18 (7.06–7.29)	6.62 (6.51–6.72)	7.01 (6.86–7.16)	6.81 (6.72–6.91)	<0.001	0.029	0.079	0.007
FINS (µIU/mL)	8.97 (8.77–9.16)	7.93 (7.57–8.29)	8.85 (8.48–9.21)	7.69 (7.49–7.89)	<0.001	<0.001	0.558	0.256
2 hINS (µIU/mL)	40.24 (38.72–41.76)	30.03 (27.89–32.16)	42.54 (40.99–44.09)	35.26 (33.86–36.66)	<0.001	<0.001	0.038	<0.001
TC (mmol/L)	4.79 (4.76–4.82)	4.63 (4.59–4.67)	4.77 (4.75–4.80)	4.67 (4.64–4.71)	<0.001	<0.001	0.293	0.084
TG (mmol/L)	1.82 (1.78–1.86)	1.62 (1.58–1.67)	1.39 (1.37–1.42)	1.42 (1.39–1.45)	<0.001	0.134	<0.001	<0.001
HDL-C (mmol/L)	1.22 (1.21–1.23)	1.27 (1.25–1.28)	1.37 (1.36–1.37)	1.34 (1.32–1.35)	<0.001	<0.001	<0.001	<0.001
LDL-C (mmol/L)	2.82 (2.79–2.85)	2.55 (2.52–2.58)	2.77 (2.74–2.79)	2.56 (2.53–2.60)	<0.001	<0.001	0.007	0.633
SBP (mmHg)	125.3 (124.8–125.9)	122.0 (121.3–122.6)	120.0 (119.6–120.4)	119.8 (119.1–120.4)	<0.001	0.523	<0.001	<0.001
DBP (mmHg)	80.5 (80.2–80.9)	78.4 (78.0–78.8)	76.0 (75.7–76.2)	75.6 (75.2–76.0)	<0.001	0.113	<0.001	<0.001
HOMA–IR (mIU·mmol/L^2^)	2.22 (2.15–2.29)	1.91 (1.81–2.00)	2.12 (2.04–2.21)	1.81 (1.75–1.87)	<0.001	<0.001	0.076	0.095
Current smoker (%)b,c	45.2 (43.6–46.8)	53.6 (51.6–55.5)	2.4 (2.1 –2.8)	3.3 (2.8–4.0)	<0.001	0.011	<0.001	<0.001
Current drinker (%)b,c	41.9 (40.4–43.4)	44.2 (42.3–46.2)	4.1 (3.7–4.6)	4.1 (3.5–4.8)	0.063	0.914	<0.001	<0.001

Abbreviations: 2 hINS, 2-hour postprandial insulin; 2 hPPG, 2-hour postprandial plasma glucose; BMI, body mass index; CI, confidence interval; DBP, diastolic blood pressure; FINS, fasting insulin; FPG, fasting plasma glucose; HDL-C, high-density lipoprotein cholesterol; HOMA-IR, Homeostasis Model Assessment – Insulin Resistance; LDL-C, low-density lipoprotein cholesterol; SBP, systolic blood pressure; TC, total cholesterol; TG, triglycerides.

a Data are expressed as mean (95% CI) unless otherwise indicated.

b Data are expressed as percentage (95% CI).

c The mean and percentage values were weighted and standardized to represent Chinese adults (aged ≥20 years) according to the Chinese population structure in 2006.

d *P* value from *t-*test for mean difference and chi-square test for proportion difference.

**Table 2:** Standardized Proportion (95% Confidence Interval) of Central Obesity, Overweight, and Obesity in Chinese Adults ≥20 Years, 2007–2008.

Population	Central Obesity^a^	Overweight^b^	Obesity^c^	JCDCG-MetS^d^
	WC ≥90 cm in men, ≥85 cm in women (n = 46,024)	BMI ≥24 to <28 kg/m^2^(n = 46,024)	BMI ≥28 kg/m^2^ (n = 46,024)	(n = 45,172)
Overall^e^	27.1 (26.4–27.8)	31.4 (30.6–32.2)	12.2 (11.7–12.7)	21.9 (21.2–22.5)
Men^f^	29.0 (27.9–30.0)	33.7 (32.6–34.8)	13.7 (12.9–14.5)	25.8 (24.8–26.9)
Women^f^	25.2 (24.3–26.2)	29.2 (28.1–30.2)	10.7 (10.1–11.4)	18.0 (17.2–18.9)
* P* value^g^	<0.001	<0.001	<0.001	<0.001
DM				
with	45.4 (42.3–48.6)	41.0 (37.8–44.2)	24.3 (21.5–27.2)	58.7 (55.6–61.8)
without	25.5 (24.7–26.2)	30.7 (29.9–31.5)	11.2 (10.7–11.7)	18.1 (17.4–18.8)
* P* value^g^	<0.001	<0.001	<0.001	<0.001
Hypertension				
with	44.4 (42.6–46.2)	39.4 (37.1–41.7)	26.0 (24.4–27.6)	45.0 (43.2–46.9)
without	20.6 (19.8–21.5)	28.3 (27.4–29.2)	7.8 (7.3–8.3)	12.8 (12.1–13.6)
* P* value^g^	<0.001	<0.001	<0.001	<0.001
Urban^e^	30.0 (29.1–30.9)	34.4 (33.4–35.3)	13.2 (12.6–13.8)	24.6 (23.7–25.5)
Men^f^	34.9 (33.5–36.3)	39.1 (37.6–40.6)	15.9 (14.8–17.0)	30.7 (29.4–32.1)
Women^f^	25.2 (24.0–26.4)	29.7 (28.5–30.9)	10.5 (9.9–11.2)	18.6 (17.6–19.7)
* P* value^g^	<0.001	<0.001	<0.001	<0.001
Rural^e^	24.7 (23.7–25.7)	28.9 (27.8–30.1)	11.3 (10.6–12.1)	19.5 (18.5–20.5)
Men^f^	24.0 (22.6–25.6)	29.1 (27.5–30.7)	11.8 (10.7–13.1)	21.5 (20.0–23.1)
Women^f^	25.3 (23.9–26.7)	28.8 (27.2–30.4)	10.9 (10.0–11.9)	17.5 (16.2–18.8)
* P* value^g^	0.252	0.779	0.240	<0.001
Sex– and age–specific				
Men, age, y				
20–29	17.5 (15.1–20.0)	22.4 (19.8–25.1)	10.3 (8.4–12.5)	12.9 (10.7–15.5)
30–39	27.9 (25.8–30.0)	34.1 (31.8–36.5)	15.1 (13.4–16.9)	21.4 (19.4–23.5)
40–49	31.5 (29.5–33.7)	38.8 (36.5–41.1)	14.4 (13.0–16.0)	28.5 (26.4–30.6)
50–59	33.6 (31.2–36.0)	37.0 (34.5–39.5)	15.6 (13.9–17.6)	31.3 (29.0–33.8)
60–69	31.8 (29.1–34.7)	35.8 (32.8–38.8)	11.6 (9.8–13.7)	33.9 (30.9–37.0)
≥70	36.0 (30.6–41.8)	32.3 (27.1–38.0)	12.9 (9.3–17.5)	36.3 (30.8–40.3)
* P* value^h^	<0.001	0.001	0.756	<0.001
Women, age, y				
20–29	8.4 (7.2–9.7)	15.0 (12.6–17.6)	3.5 (2.8–4.2)	3.0 (2.4–3.7)
30–39	13.3 (12.0–14.7)	24.5 (22.7–26.3)	7.7 (6.6–8.9)	6.0 (5.2–6.9)
40–49	24.6 (23.1–26.2)	34.6 (32.9–36.4)	13.0 (11.8–14.2)	15.8 (14.5–17.2)
50–59	38.4 (36.4–40.5)	38.1 (36.1–40.2)	15.2 (13.8–16.7)	30.3 (28.4–32.4)
60–69	42.6 (39.8–45.4)	37.7 (34.9–40.6)	14.5 (12.9–16.3)	37.6 (34.8–40.4)
≥70	48.5 (40.9–56.1)	29.2 (22.9–36.5)	15.2 (11.4–19.9)	40.9 (33.7–48.5)
* P* value^h^	<0.001	<0.001	<0.001	<0.001

Abbreviation: BMI, body mass index; WC, Waist Circumference.

a Central obesity was identified as waist circumference ≥90 cm in men and ≥85 cm.

b Overweight was identified as BMI ≥24 to <28 kg/m^2^.

c Obesity was BMI ≥28 kg/m^2^.

d The JCDCG-MetS was defined as three or more of the following abnormalities: 1. Central obesity (WC ≥90 cm for men and ≥85 cm for women); 2. Elevated TG (TG ≥1.7 mmol/L), or specific treatment for this lipid abnormality; 3. Reduced HDL-C (HDL-C <1.03 mmol/l), or specific treatment for this lipid abnormality; 4. Elevated BP (BP≥130/85 mmHg or current treatment for hypertension), or previously diagnosed hypertension; and 5. Elevated plasma glucose (FPG ≥6.1 mmol/L or 2 h PG ≥7.8 mmol/L) or previously diagnosed DM.

The percentage values were standardized by the direct method according to the Chinese population structure in 2006,

e adjusted for age and sex,

f adjusted for age.

g *P* value from multivariable logistic regression analysis (adjusted for age and the interaction of region * gender).

h *P* value for linear trend from Chi-Square test for Linear-by Linear Association.

## Results

### Demographic and Clinical Characteristics

The demographic and clinical characteristics of the 46,024 subjects by gender and region are presented in **Table 1**. Generally, men had a higher WC, TG, SBP, and DBP than women, whereas women had a higher HDL-C in both urban and rural areas (all *P*<0.001). However, the urban participants had significantly higher FINS, 2 hINS, total cholesterol, LDL-C, and HOMA-IR than the rural subjects regardless of gender (all *P*<0.001). Note that the urban participants had a significantly higher WC, BMI, TG, SBP, and DBP than rural participants in men (all *P*<0.001) but not in women.

### Prevalence of Overweight and Obesity

**Table 2** presents the standardized prevalence of central obesity, overweight, obesity, and JCDCG-MetS. The prevalence of central obesity based on JCDCG definitions was 29.0% in men and 25.2% in women. The prevalence of overweight and obesity was 33.7% and 13.7% in men, and 29.2% and 10.7% in women, respectively, according to the Chinese WGOC criteria. The prevalence of JCDCG-MetS was 25.8% in men and 18.0% in women according to the JCDCG criteria. Additionally, among the participants with diabetes and among the participants with hypertension, the standardized prevalence of central obesity, overweight, obesity, and JCDCG-MetS reached 45.4% and 44.4%, 41.0% and 39.4%, 24.3% and 26.0%, and 58.7% and 45.0%, respectively. Overall prevalence of central obesity, overweight, obesity, and JCDCG-MetS were all higher in men than in women (all *P*<0.001), and were higher in urban areas than in rural areas in men (all *P*<0.001) but not in women. Linear trends in prevalence of central obesity, overweight, obesity, and JCDCG-MetS across age groups were significant for women and for men (all *P*<0.001 for trend test), except for obesity prevalence in men.

Standardized mean BMI and waist circumference by gender and age group are shown in **Table S1**.

### Association of Separate or Combined Waist Circumference/BMI Categories with CMD and CVD

Association of WC/BMI categories with CMD and CVD are shown in **Figures 1A, 1B, **and 1C, respectively. In general, multivariate regression analysis revealed that after adjustment for the confounding factors, WC groups were at higher risk of having DM than BMI groups for the same SD increment, whereas BMI groups were at higher risk of having hypertension than WC groups; additionally, in women, increased WC had a stronger association with elevated TG and reduced HDL-C than BMI (in **Figure 1A**).

Furthermore, the total study population was divided into eight subgroups with different CMD combinations according the presence of cardiometabolic disorders, i.e. (1) the control group, without any cardiometabolic disorder (n = 11234, 27.1%); (2) the three groups with only one cardiometabolic disorder: the DM group (n = 501, 1.2%), the hypertension group (n = 2648, 6.4%), or the dyslipidemia group (n = 15915, 38.4%); (3) the three groups with two cardiometabolic disorders: the DM plus hypertension group (n = 403, 1.0%), the DM plus dyslipidemia group (n = 1529, 3.7%), and the hypertension plus dyslipidemia group (n = 7159, 17.3%); and (4) the one group with three cardiometabolic disorders: the DM plus hypertension and dyslipidemia group (n = 2104, 5.1%). Then, the associations of combinations of CMD with the BMI and WC categories were analyzed in multinomial regressions**.** The results showed that after adjustment for the confounding factors, After 1-SD increment, the WC groups were more likely to have DM plus dyslipidemia than the BMI groups (3.39 vs 1.77 for the two Group III and 4.55 vs 2.09 for the two Group IV, all *P*<0.01), whereas the BMI groups were more likely to having hypertension plus dyslipidemia than the WC groups (3.48 vs 2.70 for the two Group III and 5.88 vs 3.37 for the two Group IV, all *P*<0.01). There were no significant differences in associations of WC and BMI with the presence risk of DM plus hypertension or with the presence risk of DM plus hypertension and dyslipidemia (**Figures 1B** **)**.

Compared to the lowest group of WC/BMI, the highest BMI group (BMI ≥28 kg/m^2^) was associated with a 1.9-fold greater risk of CHD in men, and the highest WC group (WC ≥90 cm) was associated with a 2.1-fold greater risk of CHD in women, even when the effects of CMD were controlled (**Figure 1C**, Model 2).

Bar graphs of ORs for CMD and CVD among the combination groups of WC and BMI categories in all participants are shown in **Figures 2A and 2B**, respectively. Using the combination group with the lowest WC and BMI categories (WC <85 cm in men or WC <80 cm in women and BMI <24 kg/m^2^) as the referent (OR = 1)**,** the ORs of DM, hypertension, and dyslipidemia increased significantly per 0.5-SD increment in WC or in BMI after adjusting for gender plus confounding factors **(** **Figure 2A** **)**. Note that hypertension risk increased more quickly across elevated BMI than across WC. For example, the adjusted OR of hypertension in those with the highest BMI and the lowest WC categories (BMI ≥28 kg/m^2^ and WC <85 cm in men or WC <80 cm in women) was 4.1 (2.9–5.8), but was 1.8 (1.4–2.2) in those with the lowest BMI and the highest WC categories (BMI <24 kg/m^2^ and WC ≥95 cm in men and ≥90 cm in women) when compared with the referent.

In addition, when compared to the highest separate WC and BMI categories, the highest combination of WC and BMI categories (BMI ≥28 kg/m^2^ and WC ≥95 cm in men and ≥90 cm in women) was associated with greater DM, hypertension, and dyslipidemia risk. The ORs (95% CI) in all participants were 2.19 (1.96–2.44) vs 1.88 (1.67–2.12) and 1.12 (0.99–1.26); 5.70 (5.24–6.19) vs 1.51 (1.39–1.65) and 1.69 (1.57–1.82); and 3.73 (3.42–4.07) vs 2.16 (1.98–2.35) and 1.33 (1.25–1.40), respectively (data for joint WC and BMI categories shown in **Figure 2A** **;** data for separate WC and BMI categories not shown here).

With the effects of CMD controlled, the ORs of CHD and CVD significantly increased 0.9-fold and 0.5-fold, respectively, even in those combination groups with a BMI of 24–26 kg/m^2^ and WC of 90–95 cm in men or 85–90 cm in women, and the ORs (95% CIs) of stroke and CVD in the group with a BMI ≥28 kg/m^2^ and WC <85 cm in men or WC <80 cm in women increased 2.2-fold and 1.5-fold, respectively **(** **Figure 2B** **)**. Further analysis showed that six CVD cases (3 men and 3 women) were identified in this group (*n* = 191).

Figure S1 depicts receive operating characteristic curve of combined WC and BMI categories in identifying individuals with CMD or CVD and Table S2 shows that optimal cutoffs of combined WC and BMI categories were selected from the ROC curve in identifying individuals with CMD or CVD according to a larger Youden Index and better trade-off between sensitivity and specificity.

## Discussion

This report is one of a series of reports from the large 2007–2008 China National Diabetes and Metabolic Disorders Study [2], [16], [17]. Our report focuses on the prevalence of obesity and the clinical utility of separate and combined WC and BMI categories and category increments as indicators of CMD and CVD risk. The large size of the population studied gives our analyses good statistical power and broad generalizability to the Chinese adult population.

### Prevalence of Central Obesity, Overweight, and Obesity

Our results indicate that 27.1% (approximately 258.2 million) of Chinese adults are centrally obese according to the the JCDCG standards [20]. At the same time, 31.4% and 12.2% (approximately 299.5 and 116.2 million, respectively) of Chinese adults are overweight and obese according to the Chinese WGOC definition [21]. According to the same criteria, the prevalence of overweight and obesity in Chinese adults was 16.4% and 3.6% in 1992, and 22.8% and 7.1% in 2002 [25], [26]. Given the approximate 50% increase in the combined prevalence of overweight and obesity observed in the 10 years between these studies, and the additional 72% increase in obesity observed in our study. Note that the prevalence of central obesity raised about 1.7 times among participants with diabetes, and general obesity raised about 2.1-fold among the participants with hypertension compared to that of the general population. Our results also show that obesity and its health consequences are, and continue to be, a serious threat to public health in China [8], [27]. Obesity prevention needs to be actively implemented.

### Separate Impact of WC and BMI on CMD and CVD

The relationship of WC/BMI with CMD and CVD vary in a variety of epidemiologic studies, depending on the sample size, the sample’ representativeness, the scale for measurement of variables, and the confounding factors considered. Our results show that, after a 1-SD increment, WC is associated with a 1.7-fold or 2.2-fold greater risk of having DM or DM plus dyslipidemia than BMI, while BMI was associated with a 2.3-fold or 1.7-fold higher hypertension or hypertension plus dyslipidemia risk than WC. These findings are supported by previous studies [3], [28]–[32], and our results confirm the observation that WC can be used as a good indicator of visceral fat and is closely associated with diabetes, whereas BMI better reflects body volume and mass, which is associated with blood viscosity and blood volume, and is closely related to blood pressure.

Additionally, significant heterogeneity across genders was observed in association of the three lipoprotein markers with WC/BMI. Stronger associations of WC with elevated TG and reduced HDL-C than BMI were found in women but not in men. The exact mechanisms involved in these associations are unclear.

Although an established link between WC/BMI and CVD has been documented, the debate remains about whether the link is mediated through the other CMD and which anthropometric indices are most closely associated with CVD [4]–[6], [29], [32]–[39]. In our study, the highest BMI group (BMI ≥28 kg/m^2^) was more likely to be associated with CHD in men (about 1.9 times), and the highest WC group (WC ≥90 cm) was more likely to be associated with CHD in women (about 2.1 times) compared with those with the lowest BMI or WC, respectively, even after the effects of CMD were controlled. Our results show that WC and BMI predict the risk of CHD beyond that explained by DM, hypertension, or dyslipidemia.

### Impact of Combined WC and BMI Measures on CMD and CVD

Compared with previous studies [40], our results clearly elucidated the clinical advantage of using combined WC and BMI categories, which cover the cut-offs recommended by JCDCG and WGOG in identifying risk of CMD and CVD. A combination of WC and BMI categories significantly increased the power of identifying CMD risk compared to separate WC or BMI categories. For example, the highest combination of WC and BMI categories (BMI ≥28 kg/m^2^ and WC ≥95 cm in men and ≥90 cm in women) was associated with a greater risk of having DM (about 1.2 or 2.0 times), hypertension (about 3.8 or 3.4 times), and dyslipidemia (about 1.7 o 2.8 times), compared with the separate highest WC or BMI categories, respectively.

The combination of WC and BMI categories was more likely to identify individuals having CVD risk with lower WC but the highest BMI (even <85 cm in men or <80 cm in women but BMI ≥28 kg/m^2^) and lower BMI but the highest WC (BMI of 24–26 kg/m^2^ but WC ≥90 cm in men or ≥85 cm in women) than separate WC or BMI categories (WC ≥95 cm in men or ≥90 cm in women, or BMI ≥28 kg/m^2^), even when controlling for the effect of CMD. We also noted that although WC reflects a measure of central fat distribution, while BMI reflects a combination of both fat mass and lean mass, the two parameters share almost the same information of obesity, r(Pearson correlation) = 0.775 (*P*<0.001), and even in their category variables, r(Spearman correlation) = 0.701 (*P*<0.001). So in Figure 2, the interactions of waist and BMI were only found in a few of combination groups of BMI and WC categories, for example, the interactions of waist and BMI for prevalence risk of DM were only found in the combination of BMI Group II* WC Group IV and of BMI Group III* WC Group IV. Meanwhile C-statistic and ROC curve for the interaction between waist and BMI are almost superimposable (Figure S1 and Table S2).

### Study Strengths and Weakness

This study has notable strengths. First, the large size of the population studied gives our analyses good statistical power and broad generalizability to the Chinese adult population. Secondly, taking the measurement scale and cut-offs recommended for Chinese adults into consideration, we evaluated the utility of separate and combined WC and BMI categories across matched increments in identifying risk of CMD and CVD.

Limitations of this study included oversampling of urban residents and a lower response rate among men than women. In view of this, all BMI and WC means and obesity prevalence figures were weighted to represent Chinese adults (≥20 years old) based on Chinese population data in 2006. Note taht a cross-sectional survey may preclude a causal relationship. In our study, in the case of participants reporting a non-fatal CVD event, the participant’s BMI and WC before or at the time of the presence of CMD or the incident CVD event is unknown. Any secondary causes of stroke (such as clotting disorders, atrial fibrillation, atrial septal defect) were not used to exclude participants.

In conclusion, central obesity, overweight, and obesity are epidemic in Chinese adults. WC and BMI add to the predictive power of each other, and a combination of the two indexes is superior to the separate indicators in identifying risk of CMD and CVD. This study shows that prevention and control of obesity in Chinese adults should be an urgent public health priority. A combination measurement of WC and BMI may help identify those who are most at risk for adverse cardiometabolic and cardiovascular outcomes. We also found that the cut-offs of WC and BMI recommended by Chinese JCDCG and WGOC are of clinical significance for this population.

## Supporting Information

Figure S1 **Receive operating characteristic curve of combined WC and BMI categories in identifying individuals with CMD or CVD.** Receive operating characteristic (ROC) curve analyses were conducted to determine the optimal cutoffs of combined WC and BMI categories in identifying individuals with DM, hypertension, dyslipidemia (in Figure S1A), CHD, stroke, or CVD (in Figure S1B). Area under curve (AUC) and their corresponding 95% confidence interval (CI) were presented.
(TIF)

Table S1 **Standardized Mean of BMI and Waist Circumference by Gender and Age Group.**
(DOC)

Table S2 **The optimal cutoffs of combined WC and BMI categories in identifying CMD determined by ROC curve analyses.**
(DOC)

Appendix S1 **China National Diabetes and Metabolic Disorders Study Group.**
(DOC)
