# Supplementary figures and images for: Impact of Waist Circumference and Body Mass Index on Risk of Cardiometabolic Disorder and Cardiovascular Disease in Chinese Adults: A National Diabetes and Metabolic Disorders Survey
Source: PLoS One. 2013 Mar 8;8(3):e57319. doi: 10.1371/journal.pone.0057319 (PMC3592870; doi:10.1371/journal.pone.0057319)

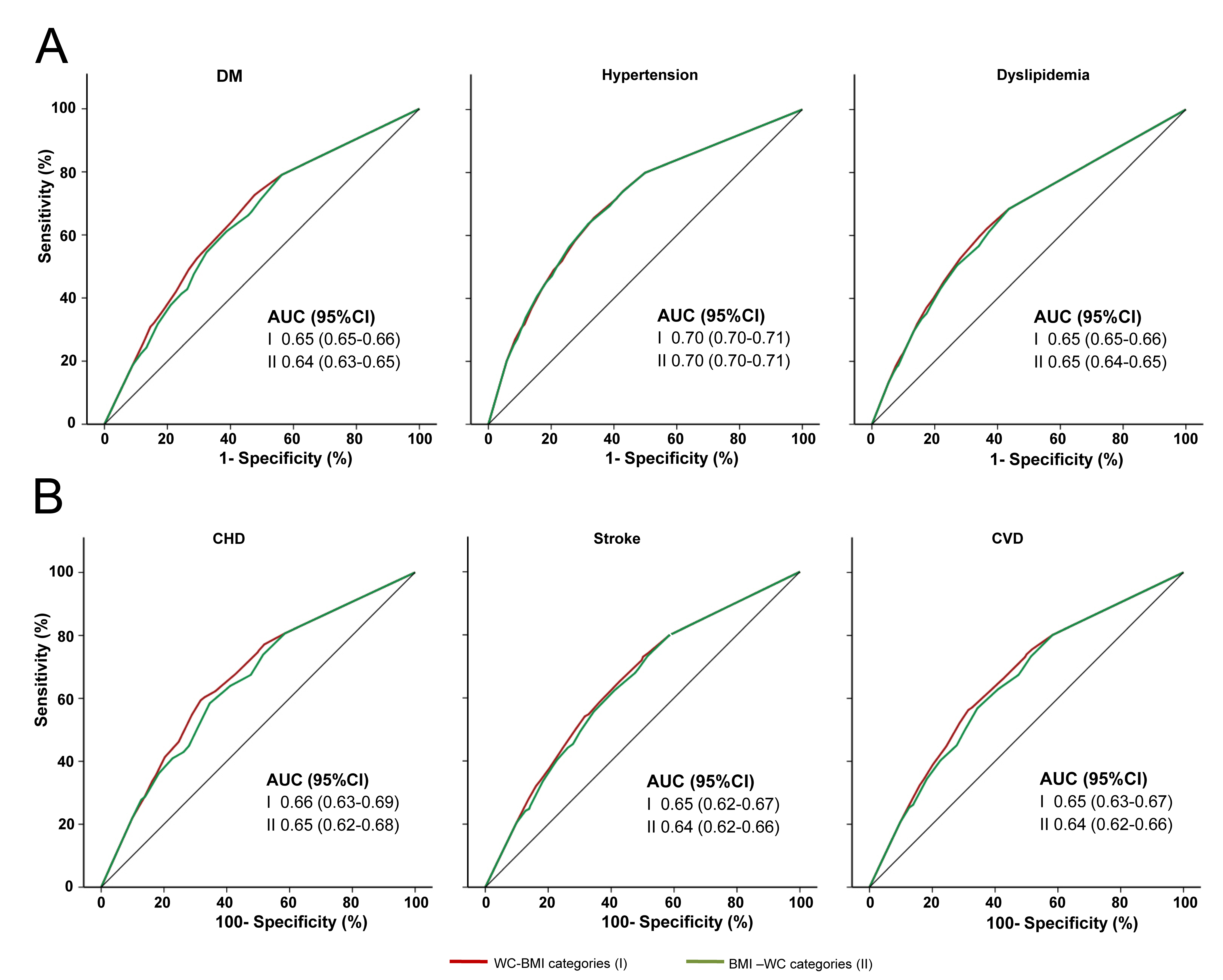

Supplement: Figure S1 — Receive operating characteristic curve of combined WC and BMI categories in identifying individuals with CMD or CVD. Receive operating characteristic (ROC) curve analyses were conducted to determine the optimal cutoffs of combined WC and BMI categories in identifying individuals with DM, hypertension, dyslipidemia (in Figure S1A), CHD, stroke, or CVD (in Figure S1B). Area under curve (AUC) and their corresponding 95% confidence interval (CI) were presented. (TIF) [file pone.0057319.s001.tif]
