# Supplementary material for: Impact of Waist Circumference and Body Mass Index on Risk of Cardiometabolic Disorder and Cardiovascular Disease in Chinese Adults: A National Diabetes and Metabolic Disorders Survey
Source: PLoS One. 2013 Mar 8;8(3):e57319. doi: 10.1371/journal.pone.0057319 (PMC3592870; doi:10.1371/journal.pone.0057319)
Supplement: Table S1 — Standardized Mean of BMI and Waist Circumference by Gender and Age Group. (DOC) [file pone.0057319.s002.doc]

**Table S1. Standardized Mean (95% Confidence Interval) of BMI and Waist Circumference by Gender and Region Type in Chinese Adults ≥20 Years, 2007-2008**

| **Population** | **n** | **Mean (95% CI)^a^ for Body Mass Index (kg/m^2^)** | | | **Mean (95% CI)^a^ for Waist Circumference (cm)** | | |
| --- | --- | --- | --- | --- | --- | --- | --- |
|  |  | **Urban** | **Rural** | **Total** | **Urban** | **Rural** | **Total** |
| Age-adjusted^b^ |  |  |  |  |  |  |  |
| Overall^c^ | 46,024 | 24.0 (23.9, 24.1) | 23.5 (23.4, 23.6) | 23.7 (23.7, 23.8) | 81.7 (81.5, 81.9) | 79.9 (79.6, 80.1) | 80.7 (80.5, 80.9) |
| Men^b^ | 18,326 | 24.6 (24.5, 24.7) | 23.6 (23.4, 23.7) | 24.0 (23.9, 24.1) | 85.5 (85.2, 85.8) | 81.8 (81.4, 82.2) | 83.5 (83.2, 83.7) |
| Women^b^ | 27,698 | 23.5 (23.4, 23.6) | 23.4 (23.2, 23.5) | 23.4 (23.3, 23.5) | 77.9 (77.7, 78.2) | 78.0 (77.7, 78.3) | 78.0 (77.8, 78.2) |
| *P* value^d^ |  | <0.001 | 0.026 | <0.001 | <0.001 | <0.001 | <0.001 |
| Sex- and age-specific |  |  |  |  |  |  |  |
| Men, age, y |  |  |  |  |  |  |  |
| 20-29 | 3086 | 23.3 (23.0, 23.7) | 22.5 (22.1, 22.9) | 22.9 (22.7, 23.2) | 80.8 (80.1, 81.5) | 78.2 (76.9, 79.4) | 79.4 (78.7, 80.2) |
| 30-39 | 3970 | 24.7 (24.5, 24.8) | 24.0 (23.7, 24.3) | 24.3 (24.1, 24.5) | 85.2 (84.6, 85.7) | 82.3 (81.4, 83.2) | 83.6 (83.1, 84.2) |
| 40-49 | 4328 | 24.9 (24.7, 25.1) | 23.9 (23.7, 24.2) | 24.4 (24.2, 24.5) | 86.6 (86.1, 87.2) | 82.9 (82.1, 83.7) | 84.6 (84.1, 85.1) |
| 50-59 | 3731 | 25.1 (24.9, 25.4) | 23.8 (23.5, 24.0) | 24.4 (24.2, 24.6) | 87.4 (86.7, 88.1) | 83.0 (82.2, 83.8) | 85.0 (84.4, 85.5) |
| 60-70 | 2311 | 24.9 (24.6, 25.1) | 23.2 (22.8, 23.5) | 23.9 (23.7, 24.2) | 87.3 (86.6, 88.1) | 81.3 (80.3, 82.3) | 84.0 (83.3, 84.7) |
| ≥70 | 900 | 24.4 (23.9, 24.9) | 23.5 (22.6, 24.4) | 23.9 (23.4, 24.4) | 86.8 (85.4, 88.1) | 82.9 (80.9, 84.8) | 84.6 (83.4, 85.9) |
| *P* value for linear trend^e^ |  | <0.001 | 0.390 | 0.017 | <0.001 | 0.001 | <0.001 |
| Women |  |  |  |  |  |  |  |
| 20-29 | 4007 | 21.4 (21.2, 21.5) | 21.5 (21.2, 21.8) | 21.4 (21.2, 21.6) | 71.4 (70.9, 72.0) | 72.0 (71.2, 72.8) | 71.7 (71.2, 72.2) |
| 30-39 | 6301 | 22.7 (22.5, 22.8) | 23.1 (22.9, 23.3) | 22.9 (22.8, 23.0) | 74.5 (74.1, 74.8) | 75.4 (74.9, 76.0) | 75.0 (74.6, 75.3) |
| 40-49 | 7058 | 24.1 (23.9, 24.2) | 24.3 (24.1, 24.5) | 24.2 (24.1, 24.3) | 78.6 (78.2, 79.0) | 79.2 (78.7, 79.7) | 78.9 (78.6, 79.2) |
| 50-59 | 6127 | 24.8 (24.6, 25.0) | 24.2 (23.9, 24.5) | 24.5 (24.3, 24.6) | 82.2 (81.7, 82.7) | 81.2 (80.5, 81.9) | 81.7 (81.2, 82.1) |
| 60-70 | 3202 | 25.1 (24.8, 25.3) | 23.5 (23.2, 23.9) | 24.2 (24.0, 24.5) | 84.3 (83.7, 84.9) | 81.1 (80.2, 82.1) | 82.6 (82.0, 83.2) |
| ≥70 | 1003 | 24.0 (23.5, 24.6) | 23.8 (23.1, 24.5) | 23.9 (23.4, 24.4) | 83.5 (81.5, 85.5) | 84.7 (82.5, 86.9) | 84.2 (82.6, 85.7) |
| *P* value for linear trend^e^ |  | <0.001 | <0.001 | <0.001 | <0.001 | <0.001 | <0.001 |

^a^Mean values were standardized by the direct method according to the Chinese population structure in 2006, ^b^adjusted for age, ^c^adjusted for age and gender.

^d^*P* value from *t-*test for mean difference.

^e^Linear trends for sex- and age-specific mean were tested using ANOVA linear test (polynomial).
