# Supplementary material for: Impact of Waist Circumference and Body Mass Index on Risk of Cardiometabolic Disorder and Cardiovascular Disease in Chinese Adults: A National Diabetes and Metabolic Disorders Survey
Source: PLoS One. 2013 Mar 8;8(3):e57319. doi: 10.1371/journal.pone.0057319 (PMC3592870; doi:10.1371/journal.pone.0057319)
Supplement: Table S2 — The optimal cutoffs of combined WC and BMI categories in identifying CMD determined by ROC curve analyses. (DOC) [file pone.0057319.s003.doc]

**Table S2A.The optimal cutoffs of combined WC and BMI categories in identifying CMD determined by ROC curve analyses.**

| **Categories** | **DM** | | | **Hypertension** | | | **Dyslipidemia** | | |
| --- | --- | --- | --- | --- | --- | --- | --- | --- | --- |
|  | **Sensitivity** (%) | **Specificity** (%) | **Youden index** | **Sensitivity** (%) | **Specificity** (%) | **Youden index** | **Sensitivity** (%) | **Specificity** (%) | **Youden index** |
| **WC-BMI categories** | 1.00 | 0.00 | 0.00 | 1.00 | 0.00 | 0.00 | 1.00 | 0.00 | 0.00 |
| WC Group I & BMI Group I | 0.79 | 0.44 | 0.23 | 0.80 | 0.50 | 0.30 | 0.68 | 0.56 | 0.25 |
| WC Group I & BMI Group II | 0.74 | 0.51 | 0.25 | 0.74 | 0.57 | 0.31 | **0.62** | **0.63** | **0.26** |
| WC Group I & BMI Group III | 0.73 | 0.52 | 0.25 | 0.72 | 0.59 | 0.31 | **0.60** | **0.65** | **0.25** |
| WC Group I & BMI Group IV | 0.72 | 0.53 | 0.25 | 0.72 | 0.59 | 0.31 | **0.60** | **0.66** | **0.25** |
| WC Group II & BMI Group I | **0.64** | **0.60** | **0.24** | **0.66** | **0.66** | **0.32** | 0.53 | 0.72 | 0.25 |
| WC Group II & BMI Group II | 0.57 | 0.66 | 0.23 | 0.58 | 0.72 | 0.31 | 0.45 | 0.77 | 0.22 |
| WC Group II & BMI Group III | 0.54 | 0.69 | 0.23 | 0.54 | 0.75 | 0.29 | 0.41 | 0.80 | 0.21 |
| WC Group II & BMI Group IV | 0.53 | 0.71 | 0.23 | 0.52 | 0.76 | 0.28 | 0.40 | 0.80 | 0.20 |
| WC Group III & BMI Group I | 0.49 | 0.73 | 0.22 | 0.49 | 0.79 | 0.28 | 0.37 | 0.83 | 0.20 |
| WC Group III & BMI Group II | 0.42 | 0.77 | 0.19 | 0.43 | 0.83 | 0.26 | 0.32 | 0.86 | 0.17 |
| WC Group III & BMI Group III | 0.36 | 0.81 | 0.17 | 0.37 | 0.86 | 0.23 | 0.26 | 0.88 | 0.14 |
| WC Group III & BMI Group IV | 0.32 | 0.84 | 0.16 | 0.32 | 0.88 | 0.20 | 0.23 | 0.90 | 0.13 |
| WC Group IV & BMI Group I | 0.31 | 0.85 | 0.16 | 0.30 | 0.90 | 0.20 | 0.21 | 0.91 | 0.12 |
| WC Group IV & BMI Group II | 0.26 | 0.87 | 0.14 | 0.27 | 0.91 | 0.18 | 0.18 | 0.92 | 0.11 |
| WC Group IV & BMI Group III | 0.19 | 0.91 | 0.10 | 0.20 | 0.94 | 0.14 | 0.13 | 0.95 | 0.08 |
| WC Group IV & BMI Group IV | 0.00 | 1.00 | 0.00 | 0.00 | 1.00 | 0.00 | 0.00 | 1.00 | 0.00 |
| **BMI-WC categories** |  |  |  |  |  |  |  |  |  |
| BMI Group I & WC Group I | 0.79 | 0.44 | 0.23 | 0.80 | 0.50 | 0.30 | 0.68 | 0.56 | 0.25 |
| BMI Group I & WC Group II | 0.71 | 0.51 | 0.22 | 0.74 | 0.57 | 0.31 | **0.61** | **0.63** | **0.24** |
| BMI Group I & WC Group III | 0.67 | 0.53 | 0.21 | **0.71** | **0.60** | **0.31** | 0.58 | 0.65 | 0.23 |
| BMI Group I & WC Group IV | 0.66 | 0.54 | 0.21 | **0.69** | **0.61** | **0.31** | 0.57 | 0.66 | 0.23 |
| BMI Group II & WC Group I | **0.61** | **0.61** | **0.22** | **0.64** | **0.68** | **0.32** | 0.50 | 0.73 | 0.23 |
| BMI Group II & WC Group II | 0.54 | 0.68 | 0.22 | 0.56 | 0.74 | 0.31 | 0.43 | 0.78 | 0.21 |
| BMI Group II & WC Group III | 0.48 | 0.72 | 0.19 | 0.50 | 0.78 | 0.28 | 0.38 | 0.81 | 0.19 |
| BMI Group II & WC Group IV | 0.43 | 0.74 | 0.17 | 0.47 | 0.80 | 0.27 | 0.35 | 0.83 | 0.18 |
| BMI Group III & WC Group I | 0.41 | 0.75 | 0.17 | 0.45 | 0.82 | 0.27 | 0.33 | 0.84 | 0.18 |
| BMI Group III & WC Group II | 0.38 | 0.79 | 0.17 | 0.41 | 0.85 | 0.25 | 0.29 | 0.87 | 0.16 |
| BMI Group III & WC Group III | 0.32 | 0.83 | 0.15 | 0.34 | 0.88 | 0.22 | 0.24 | 0.89 | 0.13 |
| BMI Group III & WC Group IV | 0.24 | 0.87 | 0.11 | 0.27 | 0.91 | 0.18 | 0.19 | 0.92 | 0.10 |
| BMI Group IV & WC Group I | 0.24 | 0.87 | 0.11 | 0.27 | 0.91 | 0.18 | 0.18 | 0.92 | 0.10 |
| BMI Group IV & WC Group II | 0.23 | 0.88 | 0.11 | 0.25 | 0.92 | 0.17 | 0.17 | 0.93 | 0.10 |
| BMI Group IV & WC Group III | 0.19 | 0.91 | 0.10 | 0.20 | 0.94 | 0.14 | 0.13 | 0.95 | 0.08 |
| BMI Group IV & WC Group IV | 0.00 | 1.00 | 0.00 | 0.00 | 1.00 | 0.00 | 0.00 | 1.00 | 0.00 |

There are two sequences for the combination of WC and BMI: (1) first ordering WC categories then ordering BMI categories within each WC category (WC-BMI categories); (2) first ordering BMI categories then ordering WC categories within each BMI category (BMI-WC categories). The optimal cut-offs of the two combinations were selected from the ROC curve in identifying individuals with CMD according to a larger Youden Index and better trade-off between sensitivity and specificity.

**Table S2B. The optimal cutoffs of combined WC and BMI categories in identifying CVD determined by ROC curve analyses.**

| **Categories** | **CHD** | | | **Stroke** | | | **CVD** | | |
| --- | --- | --- | --- | --- | --- | --- | --- | --- | --- |
|  | **Sensitivity** (%) | **Specificity** (%) | **Youden index** | **Sensitivity** (%) | **Specificity** (%) | **Youden index** | **Sensitivity** (%) | **Specificity** (%) | **Youden index** |
| **WC-BMI categories** | 1.00 | 0.00 | 0.00 | 1.00 | 0.00 | 0.00 | 1.00 | 0.00 | 0.00 |
| WC Group I & BMI Group I | 0.81 | 0.41 | 0.22 | 0.80 | 0.42 | 0.21 | 0.80 | 0.42 | 0.22 |
| WC Group I & BMI Group II | 0.77 | 0.48 | 0.25 | 0.74 | 0.48 | 0.22 | 0.75 | 0.48 | 0.24 |
| WC Group I & BMI Group III | 0.75 | 0.50 | 0.25 | 0.73 | 0.50 | 0.23 | 0.74 | 0.50 | 0.24 |
| WC Group I & BMI Group IV | 0.75 | 0.50 | 0.25 | 0.72 | 0.50 | 0.22 | 0.73 | 0.50 | 0.24 |
| WC Group II & BMI Group I | 0.68 | 0.57 | 0.25 | **0.65** | **0.57** | **0.23** | **0.66** | **0.57** | **0.24** |
| WC Group II & BMI Group II | **0.62** | **0.64** | **0.26** | **0.59** | **0.64** | **0.22** | **0.60** | **0.64** | **0.24** |
| WC Group II & BMI Group III | **0.60** | **0.67** | **0.27** | 0.55 | 0.67 | 0.22 | **0.57** | **0.67** | **0.24** |
| WC Group II & BMI Group IV | 0.59 | 0.68 | 0.28 | 0.54 | 0.68 | 0.23 | **0.56** | **0.69** | **0.25** |
| WC Group III & BMI Group I | 0.55 | 0.71 | 0.26 | 0.50 | 0.71 | 0.21 | 0.52 | 0.71 | 0.23 |
| WC Group III & BMI Group II | 0.46 | 0.75 | 0.21 | 0.44 | 0.75 | 0.19 | 0.45 | 0.76 | 0.20 |
| WC Group III & BMI Group III | 0.41 | 0.80 | 0.21 | 0.37 | 0.80 | 0.17 | 0.39 | 0.80 | 0.19 |
| WC Group III & BMI Group IV | 0.35 | 0.83 | 0.18 | 0.34 | 0.83 | 0.16 | 0.34 | 0.83 | 0.17 |
| WC Group IV & BMI Group I | 0.34 | 0.84 | 0.17 | 0.32 | 0.84 | 0.16 | 0.32 | 0.84 | 0.16 |
| WC Group IV & BMI Group II | 0.29 | 0.86 | 0.15 | 0.28 | 0.86 | 0.14 | 0.28 | 0.86 | 0.14 |
| WC Group IV & BMI Group III | 0.22 | 0.90 | 0.12 | 0.20 | 0.90 | 0.10 | 0.21 | 0.90 | 0.11 |
| WC Group IV & BMI Group IV | 0.00 | 1.00 | 0.00 | 0.00 | 1.00 | 0.00 | 0.00 | 1.00 | 0.00 |
| **BMI-WC categories** |  |  |  |  |  |  |  |  |  |
| BMI Group I & WC Group I | 0.81 | 0.41 | 0.22 | 0.80 | 0.42 | 0.21 | 0.80 | 0.42 | 0.22 |
| BMI Group I & WC Group II | 0.74 | 0.48 | 0.22 | 0.73 | 0.48 | 0.22 | 0.73 | 0.49 | 0.22 |
| BMI Group I & WC Group III | 0.69 | 0.51 | 0.21 | 0.69 | 0.51 | 0.21 | 0.69 | 0.51 | 0.20 |
| BMI Group I & WC Group IV | 0.67 | 0.52 | 0.20 | 0.68 | 0.52 | 0.20 | 0.67 | 0.53 | 0.20 |
| BMI Group II & WC Group I | **0.64** | **0.59** | **0.23** | **0.62** | **0.59** | **0.21** | **0.63** | **0.59** | **0.22** |
| BMI Group II & WC Group II | **0.58** | **0.65** | **0.24** | 0.56 | 0.66 | 0.21 | **0.57** | **0.66** | **0.22** |
| BMI Group II & WC Group III | 0.50 | 0.70 | 0.19 | 0.49 | 0.70 | 0.19 | 0.49 | 0.70 | 0.19 |
| BMI Group II & WC Group IV | 0.45 | 0.72 | 0.17 | 0.45 | 0.72 | 0.18 | 0.45 | 0.72 | 0.17 |
| BMI Group III & WC Group I | 0.43 | 0.74 | 0.17 | 0.44 | 0.74 | 0.18 | 0.43 | 0.74 | 0.17 |
| BMI Group III & WC Group II | 0.41 | 0.77 | 0.18 | 0.40 | 0.77 | 0.18 | 0.40 | 0.77 | 0.18 |
| BMI Group III & WC Group III | 0.36 | 0.82 | 0.18 | 0.34 | 0.82 | 0.15 | 0.34 | 0.82 | 0.16 |
| BMI Group III & WC Group IV | 0.29 | 0.86 | 0.15 | 0.26 | 0.86 | 0.12 | 0.27 | 0.86 | 0.13 |
| BMI Group IV & WC Group I | 0.29 | 0.86 | 0.15 | 0.25 | 0.86 | 0.11 | 0.26 | 0.86 | 0.12 |
| BMI Group IV & WC Group II | 0.28 | 0.87 | 0.15 | 0.24 | 0.87 | 0.11 | 0.25 | 0.87 | 0.13 |
| BMI Group IV & WC Group III | 0.22 | 0.90 | 0.12 | 0.20 | 0.90 | 0.10 | 0.21 | 0.90 | 0.11 |
| BMI Group IV & WC Group IV | 0.00 | 1.00 | 0.00 | 0.00 | 1.00 | 0.00 | 0.00 | 1.00 | 0.00 |

There are two sequences for the combination of WC and BMI: (1) first ordering WC categories then ordering BMI categories within each WC category (WC-BMI categories); (2) first ordering BMI categories then ordering WC categories within each BMI category (BMI-WC categories). The optimal cut-offs of the two combinations were selected from the ROC curve in identifying individuals with CVD according to a larger Youden Index and better trade-off between sensitivity and specificity.
