# Supplementary material for: Impact of Waist Circumference and Body Mass Index on Risk of Cardiometabolic Disorder and Cardiovascular Disease in Chinese Adults: A National Diabetes and Metabolic Disorders Survey
Source: PLoS One. 2013 Mar 8;8(3):e57319. doi: 10.1371/journal.pone.0057319 (PMC3592870; doi:10.1371/journal.pone.0057319)
Supplement: Appendix S1 — China National Diabetes and Metabolic Disorders Study Group. (DOC) [file pone.0057319.s004.doc]

**Appendix S1. China National Diabetes and Metabolic Disorders Study Group**

**Consulting Members:** Kunsan Xiang, Jialun Chen, Changyu Pan, Zuzhi Fu.

**Study Group Field Centers** (all field centers contributed to this work equally):

*China-Japan Friendship Hospital, Beijing, China:* Wenying Yang (principal investigator), Jianzhong Xiao, Zhaojun Yang, Danjie Ruan (Huairou first Hospital), Yufeng Li (Pinggu Hospital), Shi Bu, Hongliang Li, Peng Wang, Xueli Liu, Zhiyun Xiao.

*PLA General Hospital, Beijing, China:* Juming Lu (principal investigator), Jingtao Dou, Nan Jin, Jing Li, Shuyu Wang, Baojing Zhang, Liguang Dong.

*The Third Affiliated Hospital, Sun Yat-sen University, Guangzhou, China*: Jianping Weng (principal investigator), Longyi Zeng, Panwei Mu, Yanhua Zhang, Qiuqiong Yu, Keyi Lin, Yan Sun, Xiaodong Xu.

*Shanghai Sixth People’s Hospital, Shanghai, China*: Weiping Jia (principal investigator), Xuhong Hou, Xiaojing Ma, Huijuan Lu, Dajing Zou (Changhai Hospital), Renming Hu (Huashan Hospital), Huilin Gu (Huayang Community Health Center), Yuhua Yao (Linfen Community Health Center), Weizhen Shen (Pengpu Community Health Center), Junbin Huang (Tianmu Community Health Center), Zhijian Pan (Anting Community Health Center)

*Peking University People’s Hospital, Beijing, China*: Linong Ji (principal investigator), Xianghai Zhou, Xueyao Han, Xiuying Zhang, Yingying Luo, Xiuqing Sun, Dong Zhao (Luhe Hospital), Huifang Xing (Mentougou Hospital), Lei Liu (Haidianqu Hospital), Xinquan Zhang (Jingyuan Hospital)

*The First Affiliated Hospital, China Medical University, Liaoning, China*: Zhongyan Shan (principal investigator), Yaxin Lai, Xiaochun Teng, Ling Shan, Jiani Wang, Liangfeng Shi, Sen Wang, Li Lu, Fengwei Jiang, Beibei Wang.

*Shanxi Province People’s Hospital, Shanxi, China*: Jie Liu (principal investigator), Ling Hu, Yuying Hou (Shan Xi Medical University).

*West China Hospital, Sichuan University, Sichuan, China*: Haoming Tian (principal investigator), Xingwu Ran, Yan Ren, Hongling Yu; the First People’s Hospital of Liangshan Yi Nationality Autonomy District, Xichan City (Lisheng Cao, Hualin Lu, Xiaohua Xie); Yulin Community Health Center of Wuhou District, Chengdu (Xilian Gao); the First Hospital of Longquyi District, Chengdu (Zhong Li).

*Xijing Hospital, Fourth Military Medical University, Shanxi, China*: Qiuhe Ji (principal investigator), Fei Chen, Yaping Zhang.

*Nanjing Drum Tower Hospital, Jiangsu, China*: Dalong Zhu (principal investigator), Yun Hu, Guoyu Tong, Ning Xu, Jinluo Cheng, Junjian Chen, Fei Wang, Jiong Pei.

*Xinjiang Uygur Autonomous Region’s Hospital, Xingjiang, China*: Jiapu Ge (principal investigator), Wan Yi, Rong Wang (Bayi Steel’s Hospital), Gang Han, Huijie Mu (Habahe County Hospital), Maimaiti Aireti ( Moyu County Hospital).

*Fujian Provincial Hospital, Fujian, China*: Lixiang Lin (principal investigator), Gang Chen, Jingxin Zhao, Shuyu Yang, Mingzhu Lin (Xiamen First Hospital), Yadong Zhang, Fengyuan Zhu (Sanming First Hosptial), Mei Tu (Longyan First Hospital), Shanghua Xu (Nanping First Hospital), Weihong Lin (Jiangle County Hospital).

*Qilu Hospital, Shandong University, Shandong, China*: Li Chen (principal investigator), Yu Sun, Xiaolin Dong (Jinan Central Hospital), Yiling Fu, Kehua Zhou, Jinbo Liu, Zhenzuo Li, Yuxin Xu, Peng Lin, Wenjuan Li.

*Peking University First Hospital, Beijing, China*: Xiaohui Guo (principal investigator), Junqing Zhang, Aimei Dong, Dongming Huang, Changchun Xue (Daxinqu Hospital), Junqing Liu (Shichahai Community Health Center).

*Henan Province People’s Hospital, Henan, China*: Zhigang Zhao (principal investigator), Guijun Qin, Yong Yan, Peiyu Yao, Qinchu Li.

*Haerbin Medical University Second Hospital, Heilongjiang, China*: Qiang Li (principal investigator), Kaiting Chen, Nannan Wu, Yan Feng, Xiaoying Liu, Guozhong Li.

*Xiangya Second Hospital, Hunan, China*: Zhiguang Zhou (principal investigator), Weili Tang, Qiong Feng, Yuju Qin, Xinwen Qiu, Diaoxiang Xiao, Aiping Qin, Bin Liao, Zifang Gao, Liang Xiang.
